# One-year resource utilisation, costs and quality of life in patients with acute respiratory distress syndrome (ARDS): secondary analysis of a randomised controlled trial

**DOI:** 10.1186/s40560-016-0178-8

**Published:** 2016-08-11

**Authors:** Joachim Marti, Peter Hall, Patrick Hamilton, Sarah Lamb, Chris McCabe, Ranjit Lall, Julie Darbyshire, Duncan Young, Claire Hulme

**Affiliations:** 1 Centre for Health Policy, Imperial College London, Praed Street, London, W2 1NY UK; 2 Edinburgh Cancer Research Centre, University of Edinburgh, Crewe Road South, Edinburgh, EH4 2XR UK; 3 Central Manchester University Hospitals NHS Foundation Trust, Manchester, UK; 4 Oxford Clinical Trials Unit, University of Oxford, Oxford, UK; 5 Department of Emergency Medicine, University of Alberta, Alberta, Canada; 6 Warwick Clinical Trials Unit, University of Warwick, Coventry, UK; 7 Nuffield Department of Clinical Neurosciences, University of Oxford, Oxford, UK; 8 Academic Unit of Health Economics, University of Leeds, Leeds, UK

## Abstract

**Background:**

The long-term economic and quality-of-life outcomes of patients admitted to intensive care unit (ICU) with acute respiratory distress syndrome are not well understood. In this study, we investigate 1-year costs, survival and quality of life following ICU admission in patients who required mechanical ventilation for acute respiratory distress syndrome.

**Methods:**

Economic analysis of data collected alongside a UK-based multi-centre randomised, controlled trial, aimed at comparing high-frequency oscillatory ventilation with conventional mechanical ventilation. The study included 795 critically ill patients admitted to ICU. Hospital costs were assessed using daily data. Post-hospital healthcare costs, patient out-of-pocket expenses, lost earnings of survivors and their carers and health-related quality of life were assessed using follow-up surveys.

**Results:**

The mean cost of initial ICU stay was £26,857 (95 % CI £25,222–£28,491), and the average daily cost in ICU was £1738 (CI £1667–£1810). Following hospital discharge, the average 1-year cost among survivors was £7523 (CI £5692–£9354). The mean societal cost at 1 year was £44,077 (£41,168–£46,985), and the total societal cost divided by the number of 1-year survivors was £90,206. Survivors reported significantly lower health-related quality of life than the age- and sex-matched reference population, and this difference was more marked in younger patients.

**Conclusions:**

Given the high costs and low health-related quality of life identified, there is significant scope for further research aimed at improving care in this in-need patient group.

**Trial registration:**

ISRCTN10416500

## Background

Intensive care units (ICU) account for an important share of hospital budgets for a relatively small volume of patients given the complex procedures involved, the use of costly technology and medications, and the involvement of highly qualified staff. Patients admitted to ICU with acute respiratory distress syndrome (ARDS), an inflammatory lung condition, are particularly resource-intensive [1–3], have high mortality and morbidity [4–8], and survivors report lower quality of life than other critically ill patients [9]. Patients with ARDS often require mechanical ventilation (MV), a particularly costly life-sustaining therapy [2]. As MV itself can cause lung injury, inflammation and even death [10], alternative ventilation strategies that better protect the lungs have been called for. Recent large trials have examined the effectiveness of high-frequency oscillatory ventilation (HFOV) as compared to conventional ventilation in patients with ARDS but have not demonstrated any improvement in short-term mortality with the use of HFOV [11, 12]. A comprehensive evaluation of these alternative therapies requires the assessment of long-term mortality outcomes and the consideration of resource use and patient quality of life [13–17]. The volume of high quality data arising from recent research in this area presents new opportunities for knowledge generation beyond the core objectives of those studies.

Following discharge, recovery is challenging for survivors of ARDS as physical and neuropsychological disabilities may persist for years [6, 17–23]. In the long run, health-related quality of life (HRQL) of the survivors of ARDS and other critically ill patients has been found to be significantly lower than for the general population [1, 9, 24–32]. To date, economic studies of patients with ARDS mostly focused on ICU and hospital costs [3, 33, 34] and few studies have examined the long-term costs among ICU survivors [31, 35, 36]. Survivors of ARDS may require on-going treatments and rehabilitation following hospital discharge [1, 19, 26] as well as extensive support from carers [6], which may lead to an important economic cost to the health sector and the society as a whole [35, 37]. The measurement and reporting of costs and quality-adjusted life years (QALYs) in ICU populations is vital to allow health service and policy advances.

In this paper, we used in-hospital trial data and follow-up questionnaires of a prospective cohort of patients with ARDS to examine 1-year healthcare utilisation and quality-of-life outcomes, enabling the calculation of costs and QALYs. The economic and quality-of-life data were collected alongside the OSCAR trial [12], a multi-centre randomised, controlled trial of HFOV as compared with conventional mechanical ventilation, conducted in England, Wales and Scotland. We assessed the economic costs and benefits within the initial year following randomisation and estimated detailed costs for various parts of the care pathway (initial ICU stay, hospital stay following first ICU discharge and post-hospital resource use) and patient groups.

## Methods

### Patient population

The data used in this study come from the OSCAR study, a multi-centre, randomised, controlled trial of HFOV as compared to conventional MV in patients with moderate-to-severe ARDS. Patients (>16 years) who were undergoing MV were recruited in different-sized ICUs from 29 hospitals across England, Wales and Scotland and were randomised to either HFOV or conventional MV. Patients were eligible if they were expected to require at least two more days of MV and met the definition of moderate or severe ARDS. Patients were excluded if they had been on ventilation for 7 days or more. Inclusion criteria for OSCAR and patient recruitment have been described in detail in Young et al. [12]. The study was approved by national ethics review committees and research governance departments at each centre. Patients or their representatives provided written informed consent.

### Data collection

Characteristics such as age, gender, ventilation prior to enrolment, physiology and other information required to assess illness severity were collected at the time of randomisation. The Acute Physiology, Age, and Chronic Health Evaluation (APACHE II) score was used to measure the severity of illness within the first 24 h after the patient was admitted to an ICU. The APACHE II score is based on several physiological measurements and pre-admission health status and ranges from 0 to 71, with higher scores corresponding to more severe illness. The baseline ratio of the partial pressure of arterial oxygen (PaO2) to the fraction of inspired oxygen (FiO2) was also recorded as a measure of the severity of ARDS. Case report forms (CRFs) were completed by the medical and nursing staff for each day a patient was in ICU. The CRFs recorded the use of antibiotics, sedatives and muscle relaxant drugs as well as information on support for organ systems. Serious adverse events and whether the patient required a chest drain or presented radiological evidence of barotrauma were also recorded. ICU discharge date and location, ICU readmissions and hospital discharge date were available.

Following hospital discharge, questionnaires were sent to surviving patients and their carers at 6 and 12 months. Patients were asked about their use of medical services during the previous 6 months, including primary- and community-based health and social services and residential inpatient stays. The questionnaires also contained questions related to the use of aids and equipment, gross loss of earnings and other major expenses. Patients’ questionnaires also recorded quality of life using the EuroQol-5D (EQ-5D) [38]. Carers’ questionnaires recorded the cost of travel to and from medical services, major expenses and loss of earnings.

### Data analysis

#### Quality of life

Patients’ HRQL was measured at 6 and 12 months using EQ-5D. Health utility weights for each patient were derived from EQ-5D responses using the standard UK specific tariffs [39]. For each time period, mean utility scores were compared to those of an age- and sex-matched reference population [39].

To obtain mean QALYs at 1 year, quality of life and survival data were combined into a single metric. Patients’ histories were partitioned into several periods from randomisation to 1 year, and a utility value was assigned to each of these periods [40]. For the ventilation period, we multiplied the average number of days ventilated by the utility weight of an unconscious patient reported in the EQ-5D scoring manual (−0.4) [38]. We then calculated the mean utility scores of survivors at 6 and 12 months using all available questionnaires and, assuming a linear change in HRQL between assessments, we calculated mean utility scores at two additional mid-points (3 and 9 months). The mean time in each period was obtained by calculating the area under the relevant Kaplan-Meier survival curve. The sum of the mean survival time in each state multiplied by the utility weights provided an estimate of the quality-adjusted survival at 1 year, expressed in QALYs.

#### Costing methodology

We used a bottom-up micro-costing approach where we assigned unit costs to volumes of resource use for each patient [41]. We analysed costs from the perspective of the health and social care sector (NHS) and from a broader societal perspective. Costing was undertaken for the various pathways of care following ICU admission: initial ICU stay, hospital stay following first ICU discharge, post-hospital NHS resource use and societal costs. ICU and hospital costs were assessed using the bottom-up approach [36, 42, 43].

Resource use associated with initial stay in ICU was assessed daily based on the number of organs supported, respiratory support, whether the patient was on renal replacement therapy, whether he/she required X-rays (to check for barotrauma) or a chest drain (pneumothorax) and use of medicines. The quantities of resource use were multiplied by their corresponding unit costs, and the sum per patient for the entire stay was calculated. The unit costs of ICU resources including cost per organ supported, radiology and the cost of pneumothorax were taken from the National Schedule of Reference Costs [44]. The costs of medicines used were taken from the British National Formulary (BNF) [45]. The daily cost of ventilation was based on a fixed and per patient cost. We assumed that ventilation machines would be used for 5 years; annual maintenance and the costs of single use circuits were also included.

Once patients were discharged from ICU, the cost of step-down and the cost of ICU readmissions were calculated using the number of days until death or discharge multiplied by the cost of the level of care required, based on various nationally available references [44, 46, 47]. If a patient was discharged to another hospital following ICU, the cost of transport was taken as that of an emergency transfer. Serious adverse events and corresponding unit costs were assessed on an individual basis. Patient-reported data on resource use were collected at 6 and 12 months. NHS resource use included further inpatient care, outpatient care, primary- and community-based care and aids and equipment provided by the NHS. The costs of attendance at medical services were calculated using national reference costs multiplied by the number of times a patient attended [44, 47]. Inpatient stays were based on the number of days admitted multiplied by the corresponding unit cost [47]. The cost of aids and equipment was taken from the NHS supply chain cost for each individual item. Costs incurred by patients and their carers included cost of travel, loss of earnings and patient out-of-pocket expenses for aids, equipment and extra expenses (home adaptations). The cost of travel to and from appointments for both carers and patients was based on the distance in miles provided in the questionnaires multiplied by the cost per petrol mile as provided by HM Revenue & Customs (HMRC) [48]. Patients and carers were asked to give the gross amount lost in earnings in the 6 months covered by each questionnaire. Cost of aids and equipment purchased by patients was directly reported in the patient questionnaire. All costs were adjusted to 2012 prices using the Hospital and Community Healthcare Services (HCHS) index published by the Personal Social Services Research Unit (PSSRU) [47]. Unit costs used in the analysis and more details on the costing strategy are provided in the supplemental appendix.

#### Outcomes

The primary outcomes were 1-year survival, quality of life and resource use. Resource use was measured in terms of duration of treatment in both the ICU and the hospital and in terms of health care and societal costs at 1 year, divided into the various care pathways, i.e. ICU stay, hospital stay and post-discharge period. These outcomes were presented for various subgroups of patients based on baseline demographic, physiological, and clinical characteristic, including ventilation strategy (HFOV or conventional ventilation). Total cost per 1-year survivor was obtained by dividing the sum of ICU, hospital, and post-hospital costs for all patients by the number of patients remaining alive 1 year post-randomisation. The 1-year incremental cost effectiveness ratio (ICER) of HFOV as compared to conventional MV was also calculated. The ICER was obtained by dividing the difference between the average 1-year costs in the HFOV group, and the average 1-year cost in the conventional ventilation group by the difference between average health outcomes (QALYs) gained in the HFOV group and those gained in the conventional ventilation group [49].

#### Statistical analyses

Patient characteristics are presented using means and standard deviations (SD) for continuous variables and proportions and percentages for categorical variables. We compared baseline characteristics of 1-year survivors and non-survivors using Student’s *t* tests for continuous variables and chi-squared tests for categorical variables. No corrections were made for multiple testing. Cost data are presented using means, 95 % confidence intervals, median and interquartile range. HRQL at 6 and 12 months and quality-adjusted survival were summarised using means and 95 % confidence intervals. HRQL of survivors was compared to population reference values using Student’s *t* tests. We first estimated mean post-hospital costs based on complete cases. To obtain total costs at 1-year for each patient, missing data on post-hospital costs among 1-year survivors was addressed by multiple imputation using chained equations. We used a truncated model in which costs were constrained to be equal or above zero and generated ten datasets. Estimates from each imputed dataset were combined following Rubin’s rule [50]. All *p* values were two-sided, and a significance level of less than 0.05 was used. R 3.0.2 and Stata 12 (Statacorp, College Station, TX, USA) were used for the analyses.

## Results

### Patients

**Fig. 1:**
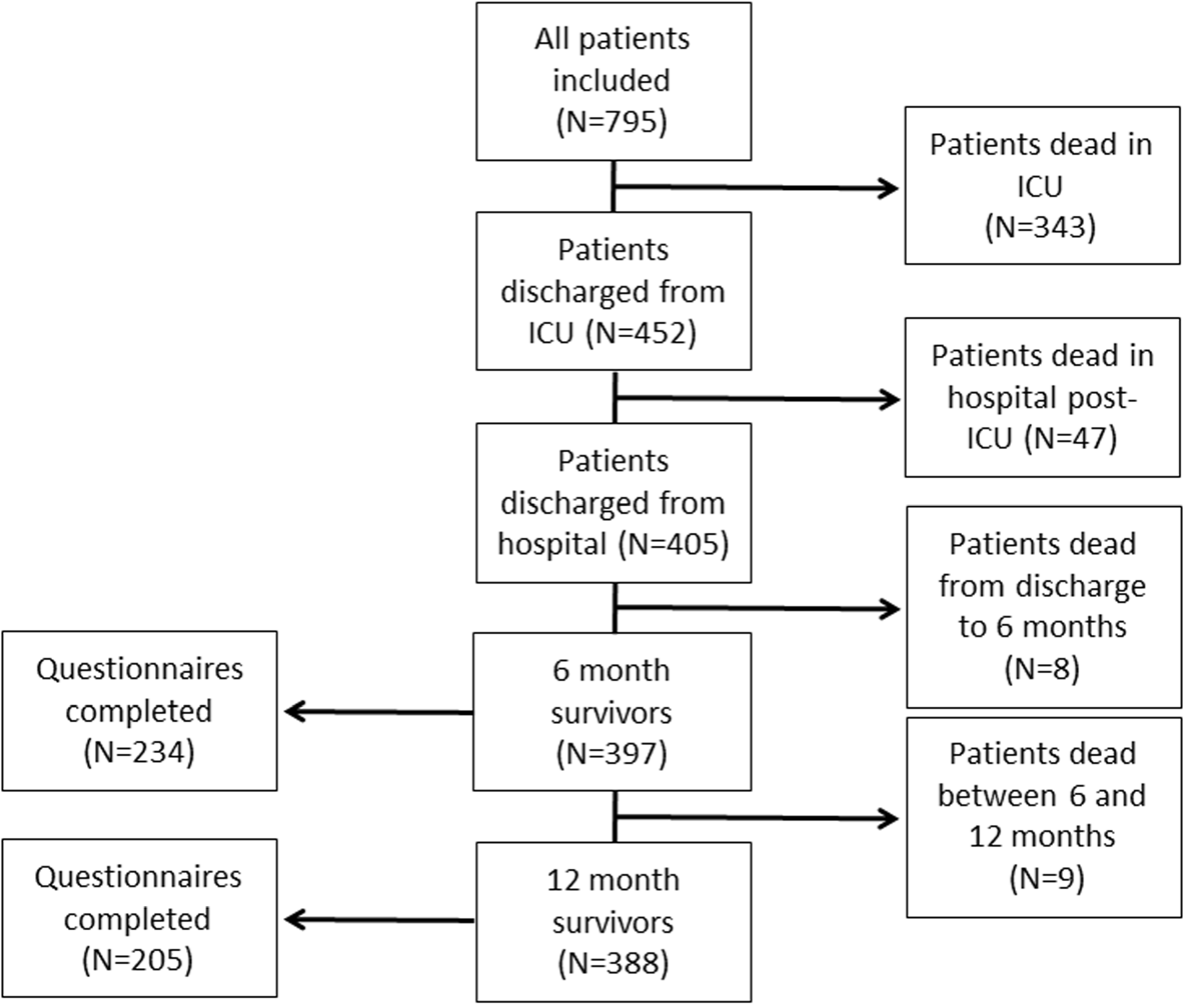
Flow chart of study population

A total of 795 patients underwent randomisation. A flow chart of the study population is presented in Fig. 1. Among the 795 patients included in the analysis, 343 patients died in ICU and 47 died in hospital after ICU discharge (in-hospital mortality rate of 49.1 %). Of the 405 remaining patients, 17 died between hospital discharge and the 12 months follow-up. Thus, the 1-year mortality rate was 51.2 %. A total of 234 patients completed the cost and quality-of-life questionnaires at 6 months, and 205 patients completed the questionnaires at 12 months (response rate of 58.9 and 52.8 %, respectively). Overall, 170 1-year survivors and 95 carers completed both the 6-month and the 12-month questionnaires. Baseline patient characteristics are displayed in Table 1. The mean age was 55 years and most patients were male (62.3 %) and had pneumonia identified as the primary cause of ARDS (58.6 %). The mean ICU length of stay (+/− SD) was 17.0+/−16.5 days, and the mean hospital length of stay was 33.6+/−43.1 days. Compared to 1-year survivors, non-survivors were older, had a higher APACHE II score, lower PaO_2_:FiO_2_ ratio and were less likely to have been admitted in surgery. 1-year survivors who have completed both 6 and 12 months questionnaires were younger and had lower APACHE II scores than 1-year survivors with incomplete information (unreported analysis).

**Table 1:** Baseline patient characteristics

Characteristic	All patients (*N* = 795)	1-year survivors (*N* = 388)	1-year non-survivors (*N* = 407)	*P* value
Age—years [SD]	55.4 [16.8]	50.2 [15.6]	60.4 [16.4]	<0.001
Female—no. (%)	300 (37.7)	148 (38.1)	152 (37.4)	0.8168
APACHE II score [SD]^a^	21.8 [6.1]	20.3 [5.7]	23.2 [6.0]	<0.001
PaO2:FiO2 ratio—KPa [SD]	15.1 [5.0]	16.0 [5.2]	14.2 [4.6]	<0.001
Prime condition: Pneumonia/pneumonitis—no. (%)	466 (58.6)	220 (56.7)	246 (60.4)	0.2849
Surgery admission—no. (%)	108 (13.6)	64 (16.5)	44 (10.8)	0.0194
ICU length of stay—days [SD]	17.0 [16.5]	19.7 [15.5]	14.4 [16.9]	<0.001
Hospital length of stay—days [SD]^b^	33.6 [43.1]	47.2 [48.6]	20.9 [32.3]	<0.001

*APACHE* Acute Physiology and Chronic Health Evaluation, *FiO2* fraction of inspired oxygen and *PaO2* partial pressure of arterial oxygen

^a^APACHE II score calculated for 765 patients only

^b^Five patients with missing information on hospital discharge

### Survival and quality of life

**Fig. 2:**
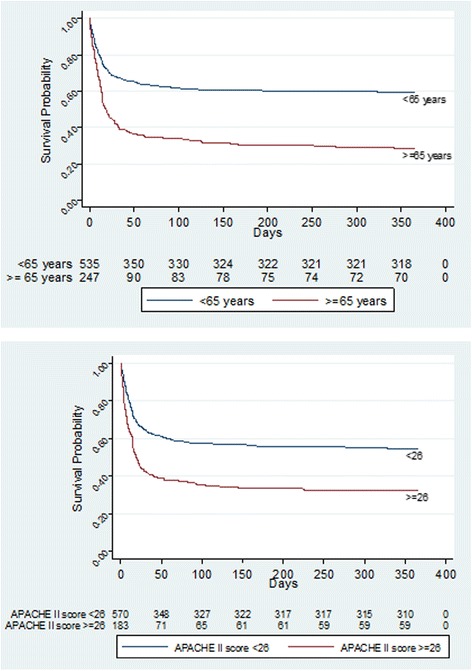
Kaplan-Meier survival plots at 12 months

Figure 2 shows the Kaplan-Meier survival estimates at 1 year by age, APACHE II score and ventilation strategy (HFOV and conventional ventilation), based on complete follow-up data on mortality. Higher 1-year mortality was observed among older (≥ 65) patients (OR 3.98, 95 % CI 2.86–5.55) and patients with higher (>26) APACHE II score (OR 2.55, 95 % CI 1.80–3.63), after adjustment for sex and PaO_2_:FiO_2_ ratio by logistic regression. The shape of the Kaplan-Meier curve suggests that the largest differences in mortality rates between these groups are observed within the first 30 days from randomisation and that, after 60 days, mortality rates were relatively low and stable in all groups. There was no significant difference in 1-year survival in the HFOV group as compared to the conventional ventilation group (OR 1.06, 95 % CI 0.79–1.43).

Health-related quality of life of survivors in the different age groups and for an age- and sex-matched population of reference is displayed in Table 2, separately for the 6 and 12 months follow-up. Survivors of ARDS reported significantly lower HRQL than the age- and sex-matched reference population and this difference was more marked in younger (<65 years) patients. Table 3 displays HRQL data at 6 and 12 months in various patient groups. Combining the survival and HRQL information, we obtained a mean quality-adjusted survival at 1 year of 0.27 (0.25–0.29), indicating that for every 100 patients admitted in ICU, 27 QALYs were generated over a 1-year period. Mean QALY over the period was significantly lower for older (≥65) patients and patients with higher (>26) APACHE II score, and higher in the HFOV group, as compared to the conventional ventilation group.

**Table 2:** HRQL of ARDS patients at 6 and 12 months, compared with age- and sex-matched reference values

	ARDS patients	Age- and sex-matched reference values	*P* value
	Mean (SD)	Mean (SD)	
EQ5D index at 6 months			
Patients under 65	0.55 (0.37)	0.85 (0.06)	<0.001
Patients 65 and above	0.62 (0.37)	0.77 (0.02)	0.003
EQ5D at 12 months			
Patients under 65	0.58 (0.35)	0.85 (0.06)	<0.001
Patients 65 and above	0.58 (0.38)	0.77 (0.02)	<0.001

**Table 3:** Quality of life

	6 months	12 months	Quality-adjusted survival at 12 months
	Mean EQ5D index (95 % CI)	Mean EQ5D index (95 % CI)	Mean QALY (95 % CI)
All patients	0.5622 (0.5163–0.6081)	0.5831 (0.5348–0.6314)	0.2676 (0.2457–0.2894)
Age			
<65	0.55 (0.49–0.60)	0.58 (0.53-0.64)	0.31 (0.28-0.35)
≥65	0.62 (0.52–0.71)	0.58 (0.48–0.68)	0.16 (0.12–0.21)
Gender			
Male	0.57 (0.51–0.63)	0.58 (0.52–0.64)	0.27 (0.23–0.30)
Female	0.55 (0.47–0.63)	0.59 (0.51–0.67)	0.27 (0.23–0.31)
APACHE II			
<26	0.57 (0.52–0.62)	0.57 (0.52–0.63)	0.29 (0.26–0.33)
≥26	0.48 (0.34–0.61)	0.60 (0.50–0.71)	0.16 (0.12–0.20)
PaO2:FiO2 ratio (KPa)			
<15	0.57 (0.51–0.64)	0.58 (0.51–0.65)	0.23 (0.20–0.27)
≥15	0.55 (0.48–0.63)	0.59 (0.52–0.65)	0.31 (0.27–0.35)
Ventilation method			
Conventional	0.52 (0.45–0.59)	0.55 (0.48–0.61)	0.25 (0.21–0.29)
HFOV	0.61 (0.54–0.67)	0.62 (0.55–0.69)	0.29 (0.25–0.33)

### Costs

As shown in Table 4, the mean per-patient cost of initial ICU stay among patients with ARDS was £26,857 (95 % CI £25,222–£28,491) and the average cost per day in ICU was £1738 (95 % CI £1667–£1810). Following initial ICU discharge, the mean cost of hospital stay among ICU survivors was £19,195 (95 % CI £15,936–£22,455) at an average daily cost of £732 (95 % CI £643–£821). Overall, a patient admitted to ICU with ARDS had an expected hospital cost of £37,626 (95 % CI £34,866–£40,385), and the average daily cost of the hospital stay was £1446 (95 % CI £1406–£1486). Following hospital discharge, the average 1-year cost amongst survivors was £7523 (95 % CI £5692–£9354). This comprised costs to the NHS (£3935), including primary and community-based care, hospital and residential stays and equipment and aids paid by the NHS, and costs to the patient and their carers (£3556), including travel costs to and from appointments, earning losses and extra expenses. These estimates were based on complete case analysis. The average 1-year post-hospital cost using multiple imputed data was £6624 (95 % CI £4464–£8784). The mean societal (total) cost at 1 year was £44,077 (95 % CI £41,168–£46,985), and the total societal cost divided by the number of 1-year survivor was £90,206 (this represents the mean 1 year cost of a survivor).

**Table 4:** Costs in different care pathways

Cost category	*N*	Mean (95 % CI)	Median	25th Centile	75th Centile
Hospital costs					
Initial ICU stay (all patients)	795				
Total cost		£26,857 (£25,222–£28,491)	£21,067	£11,654	£34,263
Daily cost		£1738 (£1667–£1810)	£1607	£1494	£1760
Post-ICU (ICU survivors)	446				
Total cost		£19,195 (£15,936–£22,455)	£7380	£2673	£20,665
Daily cost		£732 (£643–£821)	£297	£297	£1476
Hospital stay (all patients)	795				
Total cost		£37,626 (£34,866–£40,385)	£25,013	£13,991	£46,802
Daily cost		£1446 (£1406–£1486)	£1516	£1051	£1711
Post-hospital costs (1-year survivors)					
Costs to the NHS^a^	169	£3935 (£2917–£4953)	£1676	£554	£5000
Costs to the patient and their carers^b^	91	£3556 (£2332–£4780)	£395	0	£5411
Patient OOP expenses	169	£93 (£9.6–£176.4)	£0	£0	£0
Patient lost earnings	169	£2836 (£1856–£3816)	£0	£0	£2500
Carer OOP expenses	95	£565 (£305–£824)	£18	£0	£270
Carer lost earnings	93	£503 (£153–£854)	£0	£0	£0
Costs at 1 year (all patients)^c^	795	£44,077 (£41,168–£46,985)	£31,533	£20,415	£53,786
Costs at 1 year (1-year survivors)^c^	388	£54,759 (£50,357–£59,162)	£42,244	£29,170	£63,319
Cost per 1-year survivor^d^	388	£90,206 (£84,536–£96,134)			

^a^Based on 170 patients with questionnaires completed at both 6 and 12 months

^b^Based on 95 carers with questionnaires completed at both 6 and 12 months and based on 91 patients/carer questionnaires with complete information

^c^Post-hospital costs for patients with incomplete information at 6 months and/or 12 months were imputed

^d^Sum of costs among all patients, divided by the number of survivors

In-hospital resource use in patient subgroups is displayed in Table 5. Although HFOV patients had higher 1-year costs as compared to conventional ventilation patients, the difference was not statistically significant. 1-year survivors with APACHE II score over 22 exhibited higher ICU and hospital costs and had longer hospital stays than less severely ill patients. Overall, the cost of initial ICU stay accounted for 70 % of total hospital costs. As expected, healthcare costs were skewed and exhibited high between-patient variation. We identified 10 patients with extreme (>£200,000) societal costs. These patients were relatively young on average (mean 50 years) and had long hospital stays (mean 193 days).

**Table 5:** Resource use in subgroups of patients with ARDS (mean [SD])

	All patients						1-year survivors					
	*n*	ICU cost £	ICU los days	Hosp. cost £	Hosp. los days	Total societal cost^a^ £	*n*	ICU cost £	ICU los days	Hosp. cost £	Hosp. los days	Total societal cost^a^ £
Age (years)												
<65	540	28,173 [1040]	17.8 [0.72]	40,080 [1802]	35.6 [1.8]	46,882 [1817]	318	30,808 [1262]	19.9 [0.87]	47,470 [2465]	45.3 [2.50]	55,041 [2494]
≥65	255	24,070 [1381]	15.6 [1.00]	32,429 [2153]	28.6 [2.2]	38,135 [2172]	70	28,376 [2380]	19.2 [1.85]	46,175 [4023]	52.4 [4.64]	53,481 [4137]
Gender												
Male	495	25,715 [969]	16.3 [0.67]	37,017 [1780]	31.9 [1.57]	43,728 [1804]	240	28,786 [1220]	18.7 [0.85]	46,603 [2719]	45.5 [2.45]	54,425 [2771]
Female	300	28,740 [1530]	18.4 [1.09]	38,630 [2315]	35.8 [2.76]	44,652 [2323]	148	32,938 [2156]	21.5 [1.54]	48,263 [3498]	48.4 [4.26]	55,302 [3517]
APACHE II score												
<26	577	27,160 [917]	17.3 [0.63]	38,740 [1634]	35,3 [1.73]	45,171 [1647]	310	30,058 [1224]	19.7 [0.87]	47,035 [2401]	46.8 [2.5]	54,520 [2423]
≥26	188	26,167 [1909]	16.7 [1.36]	34,226 [2979]	27.9 [2.65]	40,680 [3030]	59	34,338 [3282]	21.8 [2.3]	52,160 [5985]	50.54 [5.85]	59,962 [6194]
PaO2:FiO2 ratio (KPa)												
<15	433	26,271 [1209]	16.6 [0.85]	35,518 [1897]	29.5 [1.78]	41,958 [1926]	183	32,718 [1828]	20.9 [1.3]	50,357 [3255]	45.3 [2.9]	58,010 [3292]
≥15	362	27,557 [1134]	17.8 [0.79]	40,146 [2104]	38.0 [2.28]	46,611 [2110]	205	28,273 [1340]	18.7 [0.94]	44,450 [2827]	47.8 [3.3]	51,857 [2872]
Prime condition												
Pneumonia	466	26,245 [1022]	16.7 [0.71]	36,356 [1874]	29.9 [1.6]	42,537 [1882]	220	29,987 [1380]	19.4 [0.95]	46,480 [3098]	41.7 [2.6]	53,629 [2921]
Other	329	27,723 [1410]	17.8 [1.0]	39,424 [2138]	38.3 [2.5]	46,257 [2174]	168	30,871 [1853]	20.1 [1.3]	48,227 [2849]	53.1 [3.8]	56,239 [2921]
Surgery admission												
No	687	26,883 [892]	17.1 [0.62]	37,104 [1506]	32.0 [1.5]	43,495 [1519]	324	30,700 [1194]	19.9 [0.83]	47,135 [2406]	45.2 [2.4]	54,579 [2440]
Yes	108	26,690 [2404]	17.4 [1.7]	40,942 [4015]	42.1 [4.7]	47,779 [4065]	64	28,695 [3106]	18.9 [2.26]	47,752 [4584]	53.9 [5.61]	55,672 [4636]
Ventilation method												
Conventional	397	25,606 [1110]	16.3 [0.76]	36,148 [1756]	32.5 [1.9]	42,489 [1764]	194	28,588 [1519]	18.3 [1.02]	44,343 [2499]	44.5 [2.96]	51,842 [2522]
HFOV	398	28,105 [1249]	18.0 [0.89]	39,100 [2207]	34.2 [2.1]	45,660 [2234]	194	32,151 [1639]	21.2 [1.20]	50,130 [3480]	48.7 [3.30]	57,677 [3536]
ICU los (1-year survivors)												
≤15	–	–	–	–	–	–	198	–	–	29,071 [1792]	28.7 [1.76]	36,330 [1826]
>15	–	–	–	–	–	–	190	–	–	66,166 [3468]	65.4 [3.67]	73,965 [3509]

^a^Total societal costs estimates based on imputed data for patients with missing post-hospital cost

## Discussion

In this multicentre study conducted in 29 hospitals across England, we found that adults admitted to ICU with moderate-to-severe ARDS had high 1-year mortality (51.8 %) and incurred high hospital costs (mean = £37,626; CI £34,866–£40,385). Our results are comparable to those obtained in a multi-country study where the reported daily ICU costs ranged from €1168 to €2025 [51]. However, previous ICU costing studies gave rise to a wide range of daily ICU cost estimates [51, 52], [53] due to an important variation in methodological approaches and underlying assumptions. Analyses among survivors revealed lower than normal quality of life and non-negligible resource use and costs following hospital discharge. These were equally split between health and social care costs (mean = £3935; CI £2917–£4953) and costs to the patient and their carers (mean = £3556; CI £2332–£4780). The cost per 1-year survivor was £90,206 (CI £84,536–£96,134), which is higher than figures previously published in ARDS patients [1] possibly due to higher in-hospital mortality in our sample. These figures will be of use to health economic modellers in the future when evaluating ICU interventions; especially given existing evidence for the UK is scarce. Our study revealed heterogeneity in costs across patient groups, especially regarding age and disease severity, but these results were mostly driven by higher short-term mortality in older and more severely ill patients. In line with previous studies [1, 26], we found that hospital costs accounted for the largest share of the economic burden of ARDS at 1 year (81.6 %) and that the length of ICU stay was the main cost driver. However, as previously shown [17, 37], costs can still be important following hospital discharge. Survivors required extensive community-based and social care services, specific aids and equipment and incurred extra expenses related to home adaptations. In addition, indirect costs such as lost earnings caused by employment reductions and the economic value of support from caregivers were non-negligible.

The 1-year mortality was 51.8 % in our sample, which is in line with results reported in previous studies [31]. The mean health-related quality of life of 1-year survivors was significantly lower than the age- and sex-matched reference values, and the difference was particularly marked among younger patients. When survival and quality of life were combined in the full sample, we found low quality-adjusted survival at 1 year. Specifically, we found that 100 patients admitted in ICU with ARDS are expected to accrue 27 QALYs in 1 year, in line with previous estimates [24]. Overall, high mortality combined with poor quality of life among survivors gives rise to low benefit per patient 1 year post-ICU admission and high cost per survivor.

The data used in this study were collected alongside the OSCAR trial of high-frequency oscillatory ventilation (HFOV), as compared to conventional ventilation. The clinical results of the OSCAR trial have shown no significant effect of HFOV on 30-day mortality and showed no short-term benefits or harm of HFOV [12]. In this longer term economic analysis, we found no difference in survival between the HFOV and conventional ventilation group, but we found that patients in the HFOV arm had better quality of life at 6 and 12 months and had higher average costs. A comprehensive cost-effectiveness analysis alongside the OSCAR trial was published elsewhere and found no evidence of cost-effectiveness of HFOV as compared to conventional ventilation [54].

Our study has several limitations that should be noted. First, data on the use of community-based health and social care among survivors were collected retrospectively using self-completed questionnaires, which may have affected the accuracy of the data due to reporting and recall bias [55]. As is inherent with most questionnaire-based research, our study suffered from incomplete return of the quality-of-life and resource use questionnaires. Although partially mitigated against by multiple imputation, our results may therefore be biased if the return rate depends on patient health status. Specifically, if missing data are more prevalent in sicker patients, our cost estimates are likely to be biased downwards and our quality of life estimates upwards. Also, both costs and quality of life estimates were based on a linearity assumption as we had to rely on two data collection points for their calculation. Finally, we restrained our analysis to costs and outcomes incurred over 1 year. Several studies have assessed the long-term benefits of intensive care and found that ICU was a reasonably good use of resources, especially in low-risk patients, i.e. patients with lower disease severity and higher short-term survival [31, 56, 57].

The results presented here are based on longitudinal trial data collected in a large sample of ARDS patients treated in 29 different-sized ICUs, which improves external validity. In addition, the 1-year follow-up period enabled us to capture on-going risk of death beyond the typical “short-term” follow-up periods of similar studies. Additionally, we are among the first to provide estimates of societal costs following ARDS. The estimates presented in this paper, broken down in several patient groups and care settings, will be of use to health economic modellers requiring cost and utility estimates to populate decision-analytical models [58].

## Conclusions

Given the high costs and low health-related quality of life identified, there is significant scope for further research aimed at improving care in this in-need patient group.

## Abbreviations

APACHE, Acute Physiology, Age, and Chronic Health Evaluation; ARDS, acute respiratory distress syndrome; BNF, British National Formulary; CRF, case report form; EQ-5D, EuroQol-5D; FiO2, fraction of inspired oxygen; HCHS, Hospital and Community Healthcare Services; HFOV, high-frequency oscillatory ventilation; HMRC, HM revenue & Customs; HRQL, health-related quality of life; ICER, incremental cost effectiveness ratio; ICU, intensive care unit; MV, mechanical ventilation; NHS, National Health Service; NIHR, National Institute for Health Research; PaO2, partial pressure of arterial oxygen; PSSRU, Personal Social Services Research Unit; QALY, quality-adjusted life year; SD, standard deviation
